# Protective Football Headgear and Peripheral Visuomotor Ability in NCAA Football Athletes: The Role of Facemasks and Visors

**DOI:** 10.3390/jfmk6020034

**Published:** 2021-04-08

**Authors:** Christopher G. Ballmann, Anna C. Covington, Rachel A. Miller, Rebecca R. Rogers

**Affiliations:** 1 Department of Kinesiology, Samford University, Birmingham, AL 35229, USA; acovingt@samford.edu (A.C.C.); rachel.miller.atc@gmail.com (R.A.M.); rrogers1@samford.edu (R.R.R.); 2 ATI Physical Therapy & Rehabilitation Clinics, Gardendale, AL 35071, USA

**Keywords:** tinted, target detection, reaction time, peripheral vision, dynavision

## Abstract

The purpose of this investigation was to determine the effects of varying facemask reinforcement and visor tint on peripheral visuomotor abilities in collegiate football players. Division I NCAA football players (*n* = 14) completed two peripheral visuomotor experiments: (1) Varying facemask reinforcement, (2) Varying visor tinting. In experiment 1, participants were tested under the following conditions: baseline (no helmet; BL), helmet + light (HL), helmet + medium (HM), helmet + heavy (HH), and helmet + extra heavy (HXH) reinforced facemasks. In experiment 2, participants were tested under the following conditions: baseline (no helmet; BL), helmet only (HO), helmet + clear (HCV), helmet + smoke-tinted (HSV), and helmet + mirror-tinted (HMV) visors. For each condition, a 60 s peripheral visuomotor test was completed on a Dynavision D2 visuomotor board. For experiment 1, the BL peripheral reaction time (PRT) was faster than all facemask conditions (*p* < 0.05). Furthermore, PRT was impaired with the HXH compared to HL (*p* < 0.001), HM (*p* < 0.001), and HH (*p* = 0.001). Both HH and HXH resulted in the potentiation of PRT impairments in the outermost and inferior peripheral visual areas (*p* < 0.05). In experiment 2, BL PRT was faster than all helmeted conditions (*p* < 0.05). Additionally, PRT was slower in HSV (*p* = 0.013) and HMV (*p* < 0.001) conditions compared to HO. HMV resulted in slower PRT in all peripheral areas (*p* < 0.05) while PRT was impaired only in outer areas for HSV (*p* < 0.05). Wearing protective football headgear impairs peripheral visuomotor ability. Lighter reinforced facemasks and clear visors do not appear to exacerbate impairment. However, heavier reinforced facemasks and tinted visors further decrease visuomotor performance in outer and inferior visual areas, indicating a potential need for considerations of on-field player performance and safety.

## 1. Introduction

The evolution of protective headgear in American football has been dramatic since the development of the sport. The earliest protective headgear, which primarily functioned to protect the ears with leather, was documented from the late 1890s with the first known use in a collegiate football game occurring in 1896 [1]. As larger numbers of serious injuries and deaths were being reported from the sport [2], headgear designs became more rigid, changing from solely soft leather to being reinforced with metal or steel. Football helmets became mandatory during competition for the first time at both professional and collegiate levels around the 1940s to increase player safety and attempt to prevent head/neck injuries [3]. As newer research and technology have developed, football headgear has become bulkier and now typically consists of a form of padding covered with a hard polycarbonate shell and heavy gauged metal (i.e., carbon steel, stainless steel, titanium) facemask. While helmets are primarily designed to prevent head and neck injury, football also poses risks for orbital injury or trauma. Equipping helmets with visors has been recommended to prevent orbital injuries especially in individuals with pre-existing conditions at high risk for ocular complications [4]. Although increasing the fortification and bulk of headgear has been suggested to mitigate injury, these designs have also been shown to obstruct and impede the visual field [5,6]. Since clear sight and reactive ability during gameplay is crucial for peak performance and safety [7], understanding how modern protective football headgear influences players’ ability to respond appropriately to visual stimuli surrounding them could have important implications for players’ safety and equipment rules.

Concussions represent a serious public health concern and occur across all levels of football competition [8]. Since football is a high contact sport, the likelihood of direct contact or inertial force acting on the head and neck area are high among team sports. Indeed, collegiate football players may incur over 1400 head impacts in a single season [9]. Changes in protective football headgear designs over time have been principally directed at preventing concussions and brain injuries, although many reports have shown equivocal effectiveness [10,11,12]. The newer helmet design has been shown to decrease concussion risk in collegiate players by as much as 46% [10]. Importantly, these differences remained when controlling for total head impacts whereby players wearing newer technology had approximately half the amount of concussion incidence per 100,000 hits. High school players wearing newer helmet technology have been reported to have a decreased relative and absolute risk for concussion, although injury severity did not differ when concussions occurred [12]. Still, others have reported a limited benefit or high variability of concussion prevention with new helmet designs. McGuine et al. reported that in a sample of 1332 high school football players, there was no difference in concussion prevention between different helmet brands and the date of equipment purchase [11]. Disparities in findings have been suggested to be due to a lack of control of extraneous variables, poor study design, and differences in reporting of concussion symptoms [13]. While many investigations have focused on the safety of the structure and impact resistance of helmets, few studies to date have controlled for varying helmet accessories which may influence vision and headgear functionality, leaving the need for further study.

Football helmets have been shown by multiple investigations to impair visual field and visuomotor ability [5,14,15]. Early reports on outdated football headgear showed that football helmets alter visual field and impairments of vision may be dependent on the reinforcement of facemasks [5]. Kramer et al. showed that wearing a football helmet significantly impaired hand–eye coordination compared to non-helmeted conditions [15]. Furthermore, gross reaction time has been reported to be impaired while wearing football helmets in collegiate players [14]. Recently, our lab reported that wearing a helmet with a standardized facemask significantly impaired peripheral reaction time and target detection in collegiate football players [6]. However, the addition of a clear visor to the helmet did not further exacerbate impairments. A large gap in the literature remains on how variations of facemasks and types of visor influence vision and performance. The National Collegiate Athletic Association (NCAA) currently gives 15 different examples of legal facemasks to be used in game play in their rules and interpretations guidelines [16]. Furthermore, only the use of clear visors has been approved, while all tinted visors are illegal but are allowed in rare cases with approved medical conditions. While much of these regulations have been put in place due to safety concerns for assessing head and neck injuries [17], virtually no research has been completed to elucidate whether these types of equipment impair visual reactive performance. Since previous evidence has suggested that improvements in visuomotor ability may possibly decrease concussion incidence [18], understanding how facemasks and visors influence reactive ability has important implications for player performance and safety. The purpose of this study was to investigate how varying facemask reinforcements and visor tints influence peripheral reaction time and target detection in Division I NCAA football players. We hypothesized that: (1) heavier facemask reinforcement would impair peripheral visuomotor performance to a greater degree than no helmet and lighter reinforced facemasks, and that (2) tinted visors would impair peripheral visuomotor performance to a greater degree than no helmet, helmet with no visor, and a helmet with a clear visor. Furthermore, we predicted that while wearing a helmet only would impair performance compared to no helmet, the addition of a clear visor to the helmet would have negligible effects. 

## 2. Materials and Methods

### 2.1. Participants

To determine an adequate sample size, an a priori power analysis was calculated using G*power V 3.1.9.4 software. A previous investigation on protective football headgear and reaction time from our lab showed partial η^2^ = 0.62, or a f effect size = 1.27, for PRT impairments while wearing protective football headgear [6]. Thus, the following parameters were used to calculate sample size: repeated measures ANOVA, f = 1.27, α = 0.05, and β = 0.80. This computed the minimum sample size needed as *n* = 9. To be consistent with sample sizes from previous investigations, fourteen Division I NCAA male athletes (age = 21.0 yrs ± 1.6, height = 182.1 cm ± 7.1, body mass = 101.1 kg ± 21.4, playing experience = 12.5 yrs ± 2.6) were recruited and volunteered to participate. A specialty breakdown of players included: offense (*n* = 7), defense (*n* = 6), and special teams (*n* = 1). To ensure normal or corrected-to-normal visual acuity, all participants completed a vision screening test using a Snellen eye chart while standing six meters away, which was administered by a certified athletic trainer [6]. For inclusion, participants had to have been active on a Division I NCAA football roster in the previous year, be free from concussions for six months prior, and not report any medical history of peripheral vision abnormalities or conditions. Prior to testing, participants were asked to refrain from caffeine, nicotine, and alcohol for at least 12 h and vigorous activity for 24 h prior [19]. 

### 2.2. Experiment 1: Facemask Reinforcement

**Figure 1:**
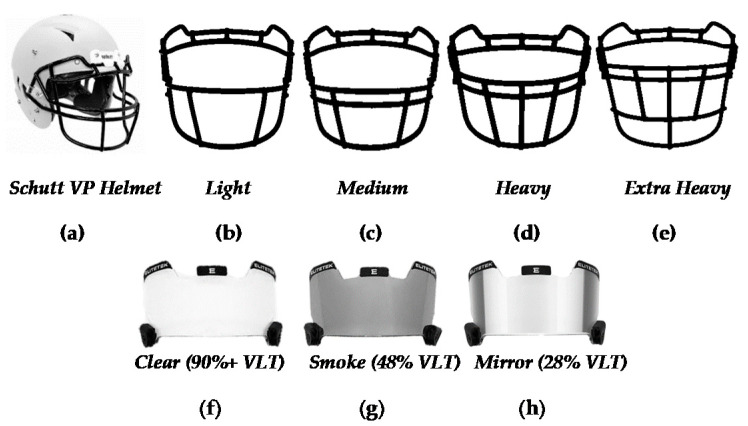
Protective football headgear: (**a**) Schutt™ Custom Vengeance Pro Helmet, (**b**) light reinforced facemask (V-ROPO-DW-TRAD), (**c**) medium reinforced facemask (V-ROPO-TRAD), (**d**) heavy reinforced facemask (V-ROPO-SW-TRAD), (**e**) extra-heavy reinforced facemask (VR JOP DW TRAD), (**f**) Elitetek clear football visor (90%+ visual light transmittance; VLT), (**g**) Elitetek smoke-tinted football visor (48% VLT), (**h**) Elitetek mirror-tinted football visor (28% VLT).

For experiment 1, participants completed a single visit consisting of peripheral visuomotor testing under these different conditions: (1) baseline with no helmet/facemask (BL), (2) helmet + facemask with light reinforcement (HL), (3) helmet + facemask with medium reinforcement (HM), (4) helmet + facemask with heavy reinforcement (HH), (5) helmet + facemask with extra heavy reinforcement (HXH). The conditions were randomized to control for possible learning effects or testing fatigue [6]. During testing, participants wore a single helmet model (Vengeance Pro LTD, Schutt; Litchfield, IL, USA) which is shown in Figure 1a. Proper helmet fitting was ensured by a certified athletic trainer prior to testing. The various facemask models (Schutt; Litchfield, IL, USA) used are shown in Figure 1b–e. Based on estimated face/neck coverage and mass, facemasks were categorized into corresponding reinforcements from NCAA permissible facemask examples [16]. Facemasks were universal and could be used on any of the various sized helmets to allow all participants to use the exact same facemasks. During testing, an exchange of facemasks was completed during rest periods.

### 2.3. Experiment 2: Visor Tint

For experiment 2, participants similarly completed an identical single visit with peripheral visuomotor testing except with differing conditions: (1) baseline with no helmet/visor (BL), (2) helmet only (HO), (3) helmet + clear visor (HCV), (4) helmet + smoke-tinted visor (HSV), and (5) helmet + mirror-tinted visor (HMV). In the same way as experiment 1, all conditions were randomized and used the same helmet model previously described for all conditions. To control for BL variations between experiments, both experiment 1 and 2 had independent BL measurements. For all helmeted conditions, a single medium reinforced facemask (Figure 1c) was used. All visors (EliteTek, Waterloo, IL, USA) used during experimentation are shown in (Figure 1f–h). For the smoke-tinted visor, visual light transmittance (VLT) was approximately 48% while the VLT for the mirror-tinted visor was approximately 28% as according to the manufacture. The clear visor allowed for near 90%+ VLT. Currently, the smoke- and mirror-tinted visors are illegal for use during gameplay in all levels of competition while the clear visor is generally allowed by most organizations/associations [16,17]. All visors were attached via quick-release clips (Under Armour, Baltimore, MD, USA). An exchange of visors was completed during rest periods in between tests.

### 2.4. Peripheral Visuomotor Testing

**Figure 2:**
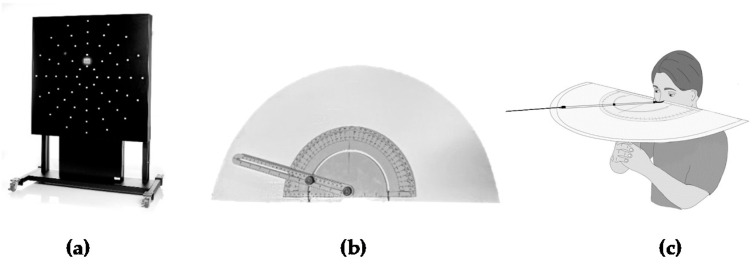
Visuomotor testing equipment: (**a**) Dynavision D2^TM^ visuomotor board (Dynavision International, West Chester, OH, USA). (**b**) Modified 180° visual protractor, (**c**) Illustration of 180° visual protractor in use. A string connected to the swing arm of the angle finder was connected to each ring to approximate visual angle/field.

For each condition in experiment 1 and 2, participants completed a 60 s peripheral visuomotor test on a Dynavision D2 visuomotor board (Axtion Technology, Palatine, IL, USA) (Figure 2a) as described previously by our lab [6]. The Dynavision D2 is equipped with 64 LED illuminated buttons situated in five concentric circles and divided into four quadrants. The D2 is height-adjustable and has been reported to be reliable and valid for assessing reaction time by multiple investigations [20,21]. The height of the board was adjusted to each participant where the tachistoscope (screen) was at eye level. The height of the board was recorded to also be used for the subsequent experiment. To determine the standing position, participants were asked to stand as far from the board as possible while being able to comfortably reach the top light of the outer fifth ring, and this distance was recorded [21]. To determine the approximate visual angle of peripheral rings for each participants’ comfortable standing distance, a visual protractor (Figure 2b,c) was as used, as others have used [22]. An angle finder (Pittsburgh Tools, Harbor Freight, Calabasas, CA, USA) was attached to a semi-circular platform which was notched to allow for nose placement at eye level. A string was attached to the end of the swing arm of the angle finder and aligned with peripheral rings 3, 4, and 5. This resulted in approximate visual angle measurements of: ring 3: 38.2 degrees ± 4.7, ring 4: 52.5 degrees ± 5.3, and ring 5: 65.5 degrees ± 4.6. These were interpreted as inner mid-peripheral (ring 3), outer mid-peripheral (ring 4), and far peripheral (ring 5) regions of peripheral vision [23]. Participants were asked to maintain their gaze forward, focused on the tachistoscope, and hit each illuminated light as quickly as possible [19]. A spirit level was attached to the side of the helmet to ensure all participants maintained a similar level starting gaze to begin each test. The peripheral visuomotor test was set up with the following parameters: 60 s test duration, proactive A mode, randomized, tachistoscope off, and activation of only outer rings (i.e., rings 3, 4, 5). In between each test/condition, participants rested for 5 min seated while equipment was prepared for the next test.

### 2.5. Statistical Analysis

All of the data analysis was completed using Jamovi software (Version 0.9; Jamovie, Sydney, Australia). To determine peripheral visuomotor ability, target detection (hit score) and the time until lighted buttons were hit (peripheral reaction time; PRT) were recorded and analyzed for each condition. PRTs were further analyzed by ring and quadrant (note: this could not be completed for hit scores due to randomized light patterns). For target detection and average PRT, a 1 × 5 [test × condition] repeated measures ANOVA was used to detect statistical differences. To detect differences by ring, a 3 × 5 [ring × condition] repeated measures ANOVA was used. For upper and lower quadrant analysis, a 2 × 5 [quadrant × condition] repeated measures ANOVA was used to detect differences. A Tukey post-hoc analysis was used for pairwise comparisons as needed to further describe significance. Estimates of the effect size for the main effects were calculated via eta squared (η^2^). Significance was set at *p* ≤ 0.05 prior to data collection. All data are presented as the mean ± standard deviation (SD).

## 3. Results

### 3.1. Experiment 1: Facemask Reinforcement

**Figure 3:**
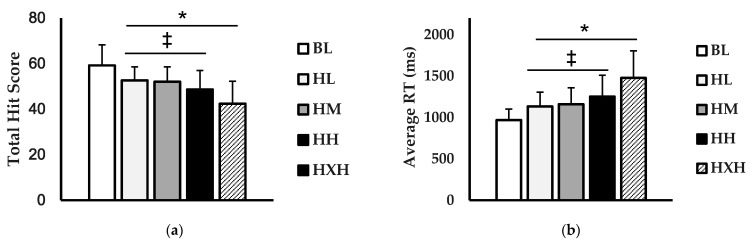
Comparison of (**a**) hit score, (**b**) peripheral reaction time (ms) between baseline (BL; white), helmet + light facemask (HL; light grey), helmet + medium facemask (HM; grey), helmet + heavy (HH; black), and helmet + extra heavy (HXH; black striped) conditions. Data are presented as mean ± SD. * indicates significant difference from baseline (*p* < 0.05). ‡ indicates significant difference from HXH (*p* < 0.05).

For experiment 1, the peripheral target detection (hit score) and peripheral vision reaction time (PRT) are shown in (Figure 3). For the hit score (hits) (Figure 3a), there was a main effect for condition (*p* < 0.001; η^2^ = 0.341). The average number of hits were higher for BL compared to HL (*p* = 0.004), HM (*p* = 0.001), HH (*p* < 0.001), and HXH (*p* < 0.001). Furthermore, the average number of hits for HL (*p* < 0.001), HM (*p* < 0.001), and HH (*p* = 0.019) was significantly higher than HXH. For the PRT (ms) (Figure 3b), there was also a main effect for condition (*p* < 0.001; η^2^ = 0.394). The PRT was faster during the BL condition compared to HL (*p* = 0.010), HM (*p* = 0.002), HH (*p* < 0.001), and HXH (*p* < 0.001). Additionally, the PRTs for HL (*p* < 0.001), HM (*p* < 0.001), and HH (*p* = 0.003) were significantly faster than HXH.

**Figure 4:**
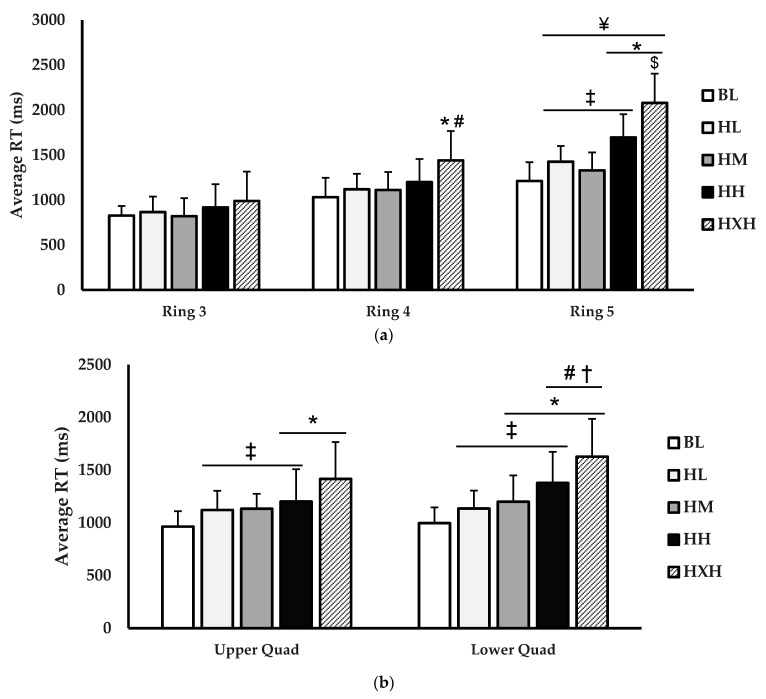
Comparison of (**a**) reaction time (ms) separated by rings (ring 3, ring 4, ring 5), (**b**) reaction time (ms) separated by upper and lower quadrants between baseline (BL; white), helmet + light facemask (HL; light grey), helmet + medium facemask (HM; grey), helmet + heavy (HH; black), and helmet + extra heavy (HXH; black striped) conditions. Data are presented as mean ± SD. * indicates significant difference from baseline (*p* < 0.05). ‡ indicates significant difference from HXH (*p* < 0.05). # indicates significant difference from HL (*p* < 0.05). † indicates significant difference from HM (*p* < 0.05). ¥ indicates significant difference from ring 3 (*p* < 0.05). $ indicated significant difference from ring 4 (*p* < 0.05).

The ring and quadrant analyses for PRT (ms) are shown in (Figure 4). For the ring analysis (Figure 4a), there was a main effect for condition (*p* < 0.001; η^2^ = 0.155) and for ring (*p* < 0.001; η^2^ = 0.341). Furthermore, there was a significant interaction for condition × ring (*p* = 0.003; η^2^ = 0.049). No significant differences existed between conditions in ring 3. In ring 4, PRT was significantly slower during the HXH condition versus BL (*p* = 0.010) and HL (*p* = 0.049). The ring 5 PRT was significantly slower during HH (*p* = 0.007) and HXH (*p* < 0.001) conditions versus BL. Furthermore, the ring 5 PRT was also slower in the HXH condition versus HL (*p* < 0.001), HM (*p* < 0.001), and HH (*p* = 0.015). When comparing ring 5 to ring 3, the PRT was slower in ring 5 during the HL (*p* < 0.001), HM (*p* < 0.001), HH (*p* < 0.001), and HXH (*p* < 0.001) compared to ring 3. The ring 5 PRT during the HXH condition was significantly slower than ring 4 for HXH (*p* = 0.002). PRT, as separated by upper and lower quadrants (Figure 4b), showed main effects for condition (*p* < 0.001; η^2^ = 0.344) but not for quadrant (*p* = 0.293; η^2^ = 0.021). No interaction for condition × quadrant (*p* = 0.218; η^2^ = 0.012) existed. For the upper quadrants, PRT was significantly slower in the HH (*p* = 0.003) and HXH (*p* < 0.001) conditions versus BL. Additionally, the PRT in upper quadrants was slower in the HXH condition compared to HL (*p* < 0.001), HM (*p* < 0.001), and HH (*p* = 0.044). In the lower quadrants, the PRT was slower during HM (*p* = 0.023), HH (*p* < 0.001), and HXH (*p* < 0.001) compared to BL. Furthermore, the lower quadrant PRT was significantly slower during the HXH condition versus HL (*p* < 0.001), HM (*p* < 0.001), and HH (*p* = 0.027). The HH (*p*= 0.003) and HXH (*p* < 0.001) lower quadrant PRT was slower than HL. Similarly, the HH (*p* = 0.048) and HXH (*p* < 0.001) lower quadrant PRT was slower than HM.

### 3.2. Experiment 2: Visor Tint

**Figure 5:**
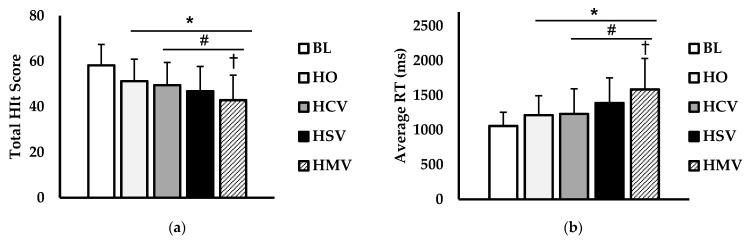
Comparison of (**a**) total hit score, (**b**) average reaction time (ms) between baseline (BL; white), helmet only (HO; light grey), helmet + clear visor (HCV; grey), helmet + smoke tinted visor (HH; black), and helmet + extra heavy (HXH; black striped) conditions. Data are presented as mean ± SD. * indicates significant difference from baseline (*p* < 0.05). # indicates significant difference from HO (*p* < 0.05). † indicates significant difference from clear (*p* < 0.05).

The peripheral target detection (hit score) and peripheral vision reaction time (PRT) for experiment 2 are shown in (Figure 5). There was a main effect for condition (*p* < 0.001; η^2^ = 0.202) for hit score (hits) (Figure 5a). The BL hit score was significantly higher than the HO (*p* < 0.001), HCV (*p* < 0.001), HSV (*p* < 0.001), and HMV (*p* < 0.001) conditions. Both the HSV (*p* = 0.011) and HMV (*p* < 0.001) conditions resulted in lower hit scores than HO. Lower hit scores during HMV compared to HCV (*p* = 0.013) were also observed. For PRT (ms) (Figure 5b), there was a main effect for condition (*p* < 0.001; η^2^ = 0.251). PRT was faster during the BL condition compared to HO (*p* = 0.020), HCV (*p* = 0.013), HSV (*p* < 0.001), and HMV (*p* < 0.001). Moreover, the PRT for the HSV (*p* = 0.014) and HMV (*p* < 0.001) conditions were slower than HO. Lower PRTs were also observed during the HMV condition versus HCV (*p* < 0.001).

**Figure 6:**
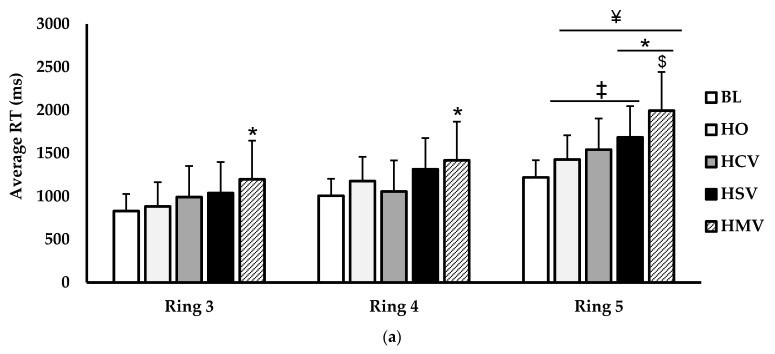

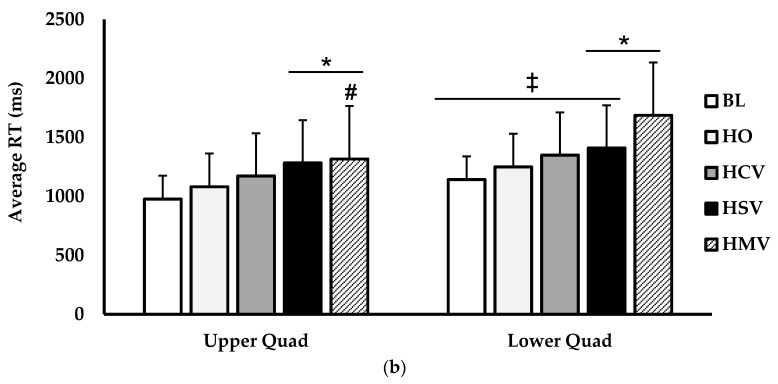
Comparison of (**a**) reaction time (ms) separated by rings (ring 3, ring 4, ring 5), (**b**) reaction time (ms) separated by upper and lower quadrants between baseline (BL; white), helmet only (H); light grey), helmet + clear visor (HCV; grey), helmet + smoke-tinted visor (HSV; black), and helmet + mirror-tinted visor (HMV; black striped) conditions. Data are presented as mean ± SD. * indicates significant difference from baseline (*p* < 0.05). # indicates significant difference from HO (*p* < 0.05). ‡ indicates significant difference from HMV (*p* < 0.05). ¥ indicates significant difference from ring 3 (*p* < 0.05). $ indicates significant difference from ring 4 (*p* < 0.05).

The ring and quadrant analyses during experiment 2 for PRT (ms) are shown in (Figure 6). For ring analysis (Figure 6a), there was a main effect for condition (*p* < 0.001; η^2^ = 0.134) and for ring (*p* < 0.001; η^2^ = 0.281). There was a significant interaction for condition × ring (*p* = 0.032; η^2^ = 0.021). In ring 3, the PRT was lower during the HMV condition versus BL (*p* = 0.004). The HMV PRT was also slower compared to BL (*p* = 0.003) in ring 4. For ring 5, the PRT was slower during the HSV (*p* < 0.001) and HMV (*p* < 0.001) conditions when compared to BL. HMV resulted in slower ring 5 reaction times compared to HO (*p* < 0.001), HCV (*p* < 0.001), and HSV (*p* = 0.044). Comparing ring 5 to ring 3, the PRT was slower in ring 5 during the HO (*p* = 0.011), HCV (*p* = 0.010), HSV (*p* < 0.001), and HMV (*p* < 0.001) versus to ring 3. PRT in ring 5 was slower than ring 4 in the HMV (*p* = 0.005) condition. Upper and lower quadrants PRTs (Figure 6b) showed a main effect for condition (*p* < 0.001; η^2^ = 0.199) and quadrant (*p* = 0.040; η^2^ = 0.088). However, there was no interaction for condition × quadrant (*p* = 0.098; η^2^ = 0.016). For the upper quadrants, PRT during HSV (*p* < 0.001) and HMV (*p* < 0.001) were significantly slower than BL. The PRT in the upper quadrants was also slower in the HMV (*p* = 0.024) condition compared to HO. The PRT in the lower quadrants was slower in the HSV (*p* = 0.005) and HMV (*p* < 0.001) conditions compared to BL. Lower quadrant PRT was also slower during the HMV condition compared to the HO (*p* < 0.001), HCV (*p* = 0.001), and HSV (*p* = 0.003) conditions.

## 4. Discussion

Evidence of protective football headgear impairing the visual field, which may be differentially altered by the type of facemask equipped on the helmet, was first described decades ago [5]. Recently, our lab showed that wearing a football helmet with a standardized facemask significantly impaired PRT and target detection compared to wearing no helmet [6]. The addition of a clear visor did not worsen reactive ability further. However, no investigations to date have explored whether various types of facemasks or visor tints influence visuomotor performance. Thus, the purpose of this investigation was to study the effects of varying facemask reinforcement and visor tint on PRT and target detection. For experiment 1, all helmeted conditions resulted in worse PRT and target hits compared to the no helmet condition regardless of facemask reinforcement. However, the extra-heavy reinforced facemask resulted in poorer PRT and hits compared to all other facemask conditions. Impairments with heavier facemask reinforcement were most prominent in far peripheral rings and lower quadrant regions. In experiment 2, all helmeted conditions resulted in worse PRT and hit scores compared to the no helmet condition, regardless of the presence of a visor. However, both the HSV and HMV conditions resulted in poorer PRT and hit scores compared to the HO condition while HMV was also worse than the HCV condition. While decrements during the HMV condition were present in all rings, declines in PRT for HSV and HMV were most pronounced in the far peripheral rings and were similarly worse in upper and lower quadrant regions. While it remains unknown how these findings translate to the field of competition, current data suggest that additions of heavier reinforced facemasks and tinted visors may negatively influence the ability to react to visual stimuli while wearing football helmets. While much more investigation is warranted to further describe how these findings may influence player safety, these data may hold critical implications for facemask and visor design along with safety guidelines.

Currently, all facemasks, regardless of reinforcement, resulted in impaired PRT and target detection compared to baseline values without a helmet. This supports our previous findings, which showed that wearing a helmet with a single facemask type impairs PRT [6]. While we cannot eliminate the possibility of the helmet structure (i.e., polycarbonate shell, padding) interfering with vision, we attribute these decrements to the presence of the facemask itself as previous studies have shown in other sport helmets without facial protection that visuomotor performance is not impaired [24,25]. Novel to the current investigation is that extra-heavy reinforcement resulted in further impairments compared to light, medium, and heavy reinforced facemasks. Visual fields have been shown to be negatively altered with football facemasks that have greater reinforcement, although in outdated headgear [5]. While varying reinforcement of football facemasks on reactive ability has not been investigated previously to our knowledge, other sports have reported poorer reactive performance while wearing heavy facemasks. For example, Dowler et al. showed that wearing a hockey helmet with a full “cage” facemask resulting in poorer response and movement times compared to a helmet alone [26]. This is bolstered by previous mechanistic evidence showing that peripheral vision restriction results in worse planning and execution of reaching and grabbing motions [27]. Pertaining to football performance, visuomotor ability and perception of motion has been linked to sports-specific skills including catching and interceptive capability [28]. Thus, the reduced visuomotor ability with heavier reinforced facemasks could possibly translate to poorer reactive ability to the ball and/or to other players on the field.

Skill positions which are reliant on catching and intercepting may be more prone to wear facemasks with lighter reinforcement. However, it is important for tight ends to be able to proficiently catch and the lineman to have the ability to catch and react to batted passes [29]. Anecdotally, the lineman and tight ends tend to wear heavier reinforced facemasks and the heavy and extra-heavy facemasks used in the current study are commonly recommended for these positions by manufactures [30,31]. The extra-heavy facemask resulted in visuomotor impairments in far areas of peripheral vision and both heavy and extra-heavy facemasks resulted in performance decrements in lower quadrants. This may have important safety implications as offensive/defensive lineman and tight ends have been reported to sustain the most knee injuries out of all positions [32]. While multiple mechanisms have been documented to contribute to these injuries, lineman and tight ends endure frequent blocks or hits below the waist, which poses serious risks for knee injury [33]. Indeed, banning of chop blocks, a technique where a player is blocked below the waist while engaged with another opponent, has been linked to lower knee injury rates in defensive players [34]. Since the heavy and extra-heavy facemasks impaired the ability to react to stimuli in lower quadrants of vision, this may result in an impaired ability to visualize and react to hits below the waist, which could possibly influence the risk of lower limb injury. Recent evidence has also shown that wearing heavier reinforced facemasks leads to increased top of the head impacts which could also influence injury to the head and neck area [35]. However, it should be cautioned that it is not well-known how varying facemask reinforcement influences injury incidence and risk necessitating future investigations to determine possible differences to enhance player safety.

Despite almost all levels of play banning the use of tinted or shaded visors, there is virtually no research on how visor tint influences visuomotor performance. Previous work from our lab showed that while wearing a football helmet impaired the peripheral reaction time, the addition of a clear visor did not cause further impairments [6]. Present data bolster those findings in that the addition of a clear visor did not worsen reaction times or target detection beyond that of wearing a helmet only in all peripheral areas and quadrants. This has also been reported in other sports whereby clear hockey visors had negligible effects on reaction and movement times [26]. However, both the smoke- and mirror-tinted visors further impaired visuomotor performance compared to solely wearing a helmet. Compared to no helmet, both the smoke- and mirror-tinted visors impaired reaction time in all quadrants while the mirrored visor had the most pronounced effects in far-peripheral areas to a greater degree than the smoke visor. We attribute the slight differences between the smoked and mirrored visors to differing visual light transmittance, although we cannot rule out the possibility of the reflective coating on the mirrored visor altering light as it passes through the material. While tinted visors and vision have not been widely studied in football, visual impairments with tinting have been noted in other contexts. Windows and windshields that are tinted have been shown to decrease depth perception and visual acuity [36]. Tinted motorcycle helmet visors have been shown to decrease visual performance compared to transparent visors [37]. Furthermore, pilots wearing helmets equipped with tinted visors were reported to have diminished dynamic visual acuity [38]. Decrements in visuomotor performance may be due to the dampening of intensity of light passing through the visor to the eye, resulting in a weaker response to stimuli. Variations in reaction times to various luminescence levels have been described for some time [39]. Evidence has shown that high-intensity visual stimuli reduces response latency and increases neural activity in parts of the brain responsible for the translation of sensory input into motor output [40]. From a practical viewpoint, this indicates that tinted visors may negatively influence players’ ability to form appropriate motor responses to visual stimuli on the field, which could be worsened even further during night games. Supporting possible poorer performance at night with tinted visors, tinted vehicle windows have been shown to diminish visual performance and target detection under night-simulated conditions [41]. The formation of appropriate reactive responses has been suggested to mitigate injury and possibly concussion in football [18]. While general safety considerations on tinted visors for the assessment of head and neck injury remain, current data suggest that reactive and visuomotor ability during gameplay may also be altered by tinted visors, which could pose additional safety concerns. However, injury risk and on-field performance were not currently measured, leaving the potential safety consequences of tinted visors during competition unknown. Further investigation is needed to determine how tinted visors affect gross reactive performance, which may translate better to gameplay and prevent injury risk.

While the current investigation presents important novel information of how protective football headgear affects peripheral visuomotor ability, findings need to be tempered as there were several limitations. First, these findings alone cannot predict how varying facemasks and visors will influence on-field performance and safety due to the controlled laboratory nature of the present experiments. Although previous investigations have used similar experimental approaches in this regard [38], most competitions will occur outdoors in the presence of UV/sunlight and other environmental factors, while the current data set was in a lumen and temperature consistent indoor space. While tinted visors impaired visuomotor ability, they may be beneficial in the presence of excess lighting on the field, although experimentally controlling lighting outside to assess this may prove difficult. Furthermore, a major drawback with visor usage during gameplay is fogging, scratches, and debris accumulating during overtime, which could not be recapitulated currently. Indeed, visor integrity has been shown to decrease over time with usage [42]. Field investigations will be needed to give greater generalizability and translation of these findings. Lastly, visors and eye shields are typically recommended for medical conditions affecting the eyes or individuals who are at risk for complications if a strike to the eye occurs. All players who participated in the current study had normal or corrected-to-normal vision and did not report any eye abnormalities. As such, caution is warranted when generalizing these findings to individuals necessitating visors due to medical concerns.

## 5. Conclusions

In conclusion, wearing a helmet, regardless of the facemask or visor type used, impairs peripheral reactive ability compared to not wearing a helmet at all. Heavier reinforced facemasks increase reaction time decrements, especially in lower areas of peripheral visual field. Furthermore, smoke- and mirror-tinted visors worsen the peripheral reactive ability beyond that of solely wearing a helmet, and this appears to be most pronounced in far-peripheral areas. To our knowledge, this is the first investigation to show that facemasks and visors differentially affect peripheral visuomotor ability. This has immense practical applications as it is plausible that these decrements in PRT and target detection may lead to compromises in both performance and player safety. Given the highly controlled laboratory setting of this investigation, a longitudinal field study is needed to determine how these findings translate to gameplay with larger sample sizes. Even with its limitations, this study may serve as a foundation for strategic examination of how helmet and facemask design may maximize player performance and safety from the perspective of visual performance. In 2011, a patent was issued for a “lateral vision football helmet” (US20110214225A1P) where clear panels formed the sidewalls in an attempt to allow for greater peripheral vision in lateral directions [43]. To our knowledge, this helmet never reached the consumer market, nor was it tested for efficacy. However, our data suggests a dire need for new headgear designs such as this, which may allow for less impedance to peripheral vision which could possibly improve safety and performance.

## Data Availability

Data are contained and available within this manuscript.
